# Non-invasive brain-spine interface: Continuous control of trans-spinal magnetic stimulation using EEG

**DOI:** 10.3389/fbioe.2022.975037

**Published:** 2022-10-31

**Authors:** Ainhoa Insausti-Delgado, Eduardo López-Larraz, Yukio Nishimura, Ulf Ziemann, Ander Ramos-Murguialday

**Affiliations:** ^1^ Institute of Medical Psychology and Behavioral Neurobiology, University of Tübingen, Tübingen, Germany; ^2^ International Max Planck Research School (IMPRS) for Cognitive and Systems Neuroscience, Tübingen, Germany; ^3^ IKERBASQUE, Basque Foundation for Science, Bilbao, Spain; ^4^ TECNALIA, Basque Research and Technology Alliance (BRTA), Donostia-San Sebastián, Spain; ^5^ Bitbrain, Zaragoza, Spain; ^6^ Neural Prosthetics Project, Department of Brain and Neuroscience, Tokyo Metropolitan Institute of Medical Science, Tokyo, Japan; ^7^ Department of Neurology and Stroke, University of Tübingen, Tübingen, Germany; ^8^ Hertie Institute for Clinical Brain Research, University of Tübingen, Tübingen, Germany

**Keywords:** brain-spine interface, EEG, trans-spinal magnetic stimulation, neuromodulation, artifact removal

## Abstract

Brain-controlled neuromodulation has emerged as a promising tool to promote functional recovery in patients with motor disorders. Brain-machine interfaces exploit this neuromodulatory strategy and could be used for restoring voluntary control of lower limbs. In this work, we propose a non-invasive brain-spine interface (BSI) that processes electroencephalographic (EEG) activity to volitionally control trans-spinal magnetic stimulation (ts-MS), as an approach for lower-limb neurorehabilitation. This novel platform allows to contingently connect motor cortical activation during leg motor imagery with the activation of leg muscles *via* ts-MS. We tested this closed-loop system in 10 healthy participants using different stimulation conditions. This BSI efficiently removed stimulation artifacts from EEG regardless of ts-MS intensity used, allowing continuous monitoring of cortical activity and real-time closed-loop control of ts-MS. Our BSI induced afferent and efferent evoked responses, being this activation ts-MS intensity-dependent. We demonstrated the feasibility, safety and usability of this non-invasive BSI. The presented system represents a novel non-invasive means of brain-controlled neuromodulation and opens the door towards its integration as a therapeutic tool for lower-limb rehabilitation.

## Introduction

Stimulation of the spinal cord constitutes a powerful technology to neuromodulate the embedded neural components responsible for controlling motor functions, such as walking. During the last decades, invasive electrical stimulation of spinal neuronal pools has been investigated in animals (Ichiyama et al., 2005; Gerasimenko et al., 2008; Alam et al., 2017) and paralyzed patients, such as stroke (Powell et al., 2022) and spinal cord injury (SCI) patients (Harkema et al., 2011; Grahn et al., 2017; Formento et al., 2018; Gill et al., 2018), to restore lower-limb motor impairment.

Magnetic stimulation of the spinal cord presents an alternative modality to neuromodulate the spinal networks non-invasively (Nardone et al., 2015). Magnetic stimulation at the spinal level can activate peripheral motor axons at their exit from the spinal cord, evoking muscle action potentials (Knikou, 2013; Matsumoto et al., 2013). There is evidence showing the capability of repetitive trans-spinal magnetic stimulation (ts-MS) to reduce spasticity in paralyzed patients improving movement (Krause et al., 2004; Beaulieu and Schneider, 2013). Furthermore, modulating spinal motor pathways through repetitive ts-MS can result in locomotor rhythms using open-loop (Gerasimenko et al., 2010) or arm-EMG-triggered protocols (Sasada et al., 2014; Nakao et al., 2015). However, these procedures lacked volitional and natural brain control.

Neural interfaces allow transferring volitional neural commands between different neuronal populations, bypassing the damaged pathways (Jackson and Zimmermann, 2012; Nishimura et al., 2013b; Kato et al., 2019). Using brain activity to control the spinal stimulation below the injury level is a natural manner of mimicking the flow of the descending commands from the brain to the spine. This phenomenon has motivated the development of brain-spine interfaces (BSIs) that aim at artificially connecting brain and spinal neural networks to recover motor function (Nishimura et al., 2013b; Zimmermann and Jackson, 2014). The BSIs record neural activity of the brain reflecting motor intentions and transform this activity into commands for spinal stimulation (Alam et al., 2016; Capogrosso et al., 2016; Yadav et al., 2020). These neural signatures associated with motor execution or motor attempt can be also detected even in patients with motor deficits (López-Larraz et al., 2015; López-Larraz et al., 2018a), which makes them suitable for BSI control. Brain-controlled spinal stimulation has been shown to be more effective than open-loop stimulation to alleviate walking impairments and to boost recovery of locomotor function (McPherson et al., 2015; Capogrosso et al., 2016; Bonizzato et al., 2018). To favor neuroplasticity, a timely linked brain activity encoding motor intention and peripheral afferent neural activation is essential (Nishimura et al., 2013a; Ramos-Murguialday et al., 2013; Mrachacz-Kersting et al., 2016; López-Larraz et al., 2018b; Kato et al., 2019). The contribution of stimulation to top-down commands due to motor intention/attempt together with the feedback from muscle contractions and afferent-evoked responses through stimulation induces an operant-conditioning effect exciting the involved neural network (brain-spine-muscles). The BSIs rely on this operating principle and the first step towards the conception of this technology must demonstrate its ability to engage the entire neural system from the brain to the muscles, both efferently and afferently.

To date, BSIs have only been developed as implantable systems, and tested in animal experiments. Non-invasive BSIs would allow broadening this field of research, facilitating experimentation in healthy subjects and patients with motor disorders. Creating a non-invasive tool would enable testing the efficacy of closed-loop *versus* open-loop ts-MS-based interventions. Electroencephalography (EEG) constitutes the most common technique for non-invasive acquisition of brain signals. However, the low signal to noise ratio and artifacts often limit EEG applications. This problem aggravates when EEG is concurrently used with electromagnetic stimulation due to signal contamination impeding the estimation of cortical activity (Insausti-Delgado et al., 2021).

In the current study, we propose an innovative design for a non-invasive BSI, relying on the continuous EEG monitoring of brain activity, removal of stimulation artifacts by median filtering, and control of the ts-MS to volitionally (but artificially) contract lower-limb muscles. To test and validate the BSI, we recruited 10 healthy participants who controlled the closed-loop system while being stimulated at 4 different conditions. As a proof of the feasibility of this interface, we report the evaluation of different indicators: *1)* neurophysiological effects of the system, *2)* usability and perception of all the users, and *3)* performance, robustness and decoding accuracy of the BSI.

## Methods

### Participants

Ten healthy participants (4 females, age = 29.5 ± 4.67 years) with no neurological disorders and complete mobility of lower limbs were recruited for the study, following previous literature sample size calculation and adjustment to pragmatism (Knikou, 2013; Sasada et al., 2014). All the participants provided written informed consent before starting the experiment. This study was approved by the Ethics Committee of the Faculty of Medicine of the University of Tübingen (Germany).

**FIGURE 1:**
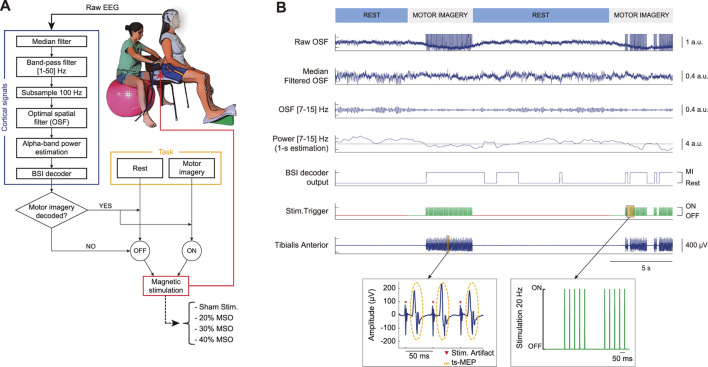
Experimental setup and functioning of the brain-spine interface (BSI) allowing simultaneous recording of cortical activity and trans-spinal magnetic stimulation. **(A)** User wearing an electrographic (EEG) system to acquire cortical activity that is processed and used to deliver contingent trans-spinal magnetic stimulation (ts-MS) over the lumbar vertebra only when motor imagery is decoded. Four conditions for the closed-loop stimulation were defined: ts-MS at 20% of the maximum stimulator output (MSO), ts-MS at 30% of the MSO, ts-MS at 40% of the MSO, and sham stimulation. **(B)** Raw EEG activity is filtered using an optimized spatial filtering (OSF) and median filtered (second trace) to remove stimulation artifacts. Sensorimotor oscillatory activity (7–15 Hz) is extracted (third trace) to compute its power and normalize it (fourth trace). When power in the alpha band decreases, falling below a predefined threshold (i.e., motor cortical activation), the BSI decodes motor imagery (fifth trace) and ts-MS is triggered at 20 Hz (sixth trace). Note that the stimulation was off during rest periods (red) and on during periods of motor imagery (green). The ts-MS pulse sends descending volleys from the spine to the tibialis anterior (TA) muscle resulting in trans-spinal motor evoked potentials (ts-MEP). The EMG trace of the TA muscle of a representative participant shows that stimulation artifacts appear approximately every 50 ms (20 Hz stimulation), while the ts-MEPs are observed ∼15 ms after the stimulation (peripheral motor conduction time from the spine to the TA).

The participants were comfortably seated on a chair, with their back straight and their right leg slightly extended, having the knee and ankle joint angles around 120° and 90°, respectively. A wedge-shaped structure was used to place the foot and ensure the angle of the leg was kept constant (Figure 1A). Neurophysiological activity was recorded by electroencephalography (EEG) and electromyography (EMG).

### Experimental design and procedure

We aimed at creating the first non-invasive brain-spine interface (BSI) that directly connects cortical activity with the modulation of spinal circuitry through lumbar stimulation and that might constitute a new technology for gait rehabilitation. Given the relevance of time-linked activation between monitored and stimulated neuronal populations, we developed a real-time system that acquires and processes EEG activity, decodes cortical activity reflecting MI of plantar dorsiflexion and triggers trans-spinal magnetic stimulation (ts-MS) (Figure 1A).

Each participant performed one session, including a screening phase and a closed-loop stimulation phase. The screening consisted of 2 blocks of 20 trials each, which included rest (10–12 s) and motor imagery (5 s) periods, each announced by an auditory cue of “Rest” and “Move”, respectively. During rest, the participants were asked to relax and stay still without executing or imagining any movement. During motor imagery (MI), the participants were asked to perform kinesthetic motor imagery of the plantar dorsiflexion of the right leg (Neuper et al., 2005). The experimenter actively inspected the quality of recording signals during the entire session to avoid physiological artifacts due to eye movements or muscle artifacts. The EEG data recorded during the screening was used to train a classifier to differentiate between the “rest” and “motor imagery” brain states.

The closed-loop stimulation phase consisted of 12 blocks of 20 trials each. We evaluated 4 stimulation conditions: 3 different ts-MS intensities and sham stimulation (further details in section *Trans-spinal magnetic stimulation*). We recorded 3 blocks of each condition, randomizing the sequence of intensities across participants, resulting in 60 trials of each condition. The number of trials was chosen based on similar studies using brain-controlled technology (Ramos-Murguialday et al., 2012; Ramos-Murguialday and Birbaumer, 2015; Naros et al., 2016, 2020). The timing of the closed-loop stimulation trials was identical to the screening trials (i.e., 10–12 s resting and 5 s of MI). Closed-loop feedback was given according to the decoded brain patterns during the MI periods (note that the stimulation was off during resting periods).

### Data acquisition

EEG activity was recorded with a commercial Acticap system (BrainProducts GmbH, Germany), with 32 channels placed on FP1, FP2, F7, F3, F4, F8, FC3, FC1, FCz, FC2, FC4, C5, C3, C1, Cz, C2, C4, C6, CP3, CP1, CPz, CP2, CP4, T7, T8, P7, P3, Pz, P4, P8, O1, and O2, following the international 10/20 system (Seeck et al., 2017). The ground and reference electrodes were located at FPz and Fz, respectively. The recording electrodes were connected to a monopolar BrainAmp amplifier (BrainProducts GmbH, Germany).

EMG activity from the right tibialis anterior (TA) muscle was recorded using Ag/AgCl bipolar electrodes (Myotronics-Noromed, Tukwila, Wa, USA) combined with an MR-compatible BrainAmp amplifier (BrainProducts GmbH, Germany). The recording electrodes had an inter-electrode space of 4 cm. The ground electrode was placed on the right patella. All the signals were synchronously acquired at 1 kHz sampling rate. Both amplifiers were directly connected to powerpacks to avoid power-line noise being introduced into the neurophysiological signals.

### Trans-spinal magnetic stimulation (ts-MS)

Due to the unnatural (and occasionally uncomfortable) sensation that the participants can experience with ts-MS, and to ensure that they were able to bear the stimulation, a familiarization session was conducted with all of them on a separate day before the experiment. We used a magnetic stimulator (Magstim Rapid2, Magstim Ltd., United Kingdom) with a circular coil (Magstim 90 mm Coil, Magstim Ltd., United Kingdom) to provide the ts-MS (biphasic single cosine cycle pulses of 400 µs).

Before starting the recording, we localized and marked the vertebrae from T12 to L5 according to anatomical landmarks. The circular coil was initially centered over the midline of the intervertebral space of T12 and shifted towards L5, advancing one vertebra in each step (coil currents directed clockwise). Single pulse stimulation was delivered above the motor threshold to locate the hot-spot of the TA muscle (i.e., the spot that led to the largest trans-spinal motor evoked potentials in 10 trials). This spot was marked for the closed-loop stimulation phase.

During the closed-loop phase, continuous brain-controlled ts-MS was applied at 20 Hz (Sasada et al., 2014). According to our previous experimental evidence, the spinal motor threshold (i.e., the minimum intensity needed for eliciting at least 5 motor evoked potentials out of 10 trials with at least 50 µV of peak-to-peak amplitude) of humans lays between 25% and 40% of the maximum stimulator output (MSO) (Insausti-Delgado et al., 2018, 2019). According to these values and aiming to encompass the most common range of human spinal thresholds, we defined 4 conditions for the closed-loop stimulation: *1)* ts-MS at 20% of the MSO, *2)* ts-MS at 30% of the MSO, *3)* ts-MS at 40% of the MSO, and *4)* sham stimulation (Figure 1A). Adding sham stimulation as control condition permits us to compare the performance of the system in the presence and absence of stimulation, and to evaluate the efficiency of the artifact elimination method. For the sham stimulation, the experimenter held the coil 1 m away from the participant, so that the stimulation took place and the participants had auditory but no sensory feedback. In this sham condition, the stimulation was set to 30% of the MSO. For the three real ts-MS conditions, the coil was placed on the hot-spot.

### Detection of movement intention

After the 2 screening blocks, a classifier was trained to discriminate between the brain states of rest and MI.

#### Data preprocessing

The EEG data were band-pass filtered with a 4th order Butterworth filter between 1 and 50 Hz. The signals were trimmed down to 15-s trials (from -10 s to +5 s with respect to the MI cue) and subsampled to 100 Hz. Optimized spatial filtering (OSF) was applied to improve the estimation of the task-related motor cortex activation. The OSF calibration consists of a gradient-descent optimization to find weights for the linear combination of electrodes that minimizes alpha power during MI and maximizes it during rest. We considered 17 electrodes (from the 32 recorded) to measure this activation: FC3, FC1, FCz, FC2, FC4, C5, C3, C1, Cz, C2, C4, C6, CP3, CP1, CPz, CP2 and, CP4. The signal of these electrodes was band-pass filtered between 7 and 15 Hz (4th order Butterworth), to isolate the modulation of the alpha rhythm, which is the target parameter of the optimization process (López-Larraz et al., 2014). This has been validated as an effective automated method to improve the measurement of event-related desynchronization (ERD) of sensorimotor activity during motor tasks (Graimann and Pfurtscheller, 2006). The result of this process is a virtual channel that synthesizes the activation over the motor cortex. The OSF weights were computed using the trials of the screening phase and kept fixed during the closed-loop phase.

#### Feature extraction

A one-second sliding window, with 200 ms sliding-step, was applied to each 15-s trial of the OSF virtual channel in the interval [−3, −1] s for the rest class and [1, 3] s for the MI class (i.e., 6 windows per class and trial). The power spectrum between 1 Hz and 50 Hz was calculated for each of these windows using a 20th-order autoregressive (AR) model with 1 Hz resolution, based on the Burg algorithm. The most discriminant range of frequencies to separate between rest and MI classes was selected by visually inspecting the signed r-squared values (point-biserial correlation coefficients). Despite alpha [(7–15) Hz] and beta [(15–30) Hz] being generally the most reactive frequency bands during MI, we restricted the selection of features to the alpha range only, to avoid the repetitive ts-MS at 20 Hz interfering with our brain features of interest. The power values within the selected frequency range were averaged, resulting in one unique feature.

All the extracted windows from the screening trials, transformed into one feature per window, were z-score normalized and used as examples of both classes to train a linear discriminant analysis (LDA) classifier.

#### Classification

During closed-loop control of the system (Figure 1B), the classifier analyzed the EEG activity in real-time and activated the stimulator when the MI brain states were detected. Cortical activity was continuously acquired in 200 ms data blocks. Every block was median-filtered (see details in section *Median filtering for ts-MS contamination removal*) based on the estimation of the median value of a 20-ms sliding window. We then implemented an OSF filter using the electrodes over the motor cortex to maximize the estimation of cortical activation (using the coefficients computed from the screening data). Due to the responsiveness of the sensorimotor activity in the alpha band (7–15) Hz during a motor task, we designed the system to identify cortical patterns within this frequency band. We appended the filtered block to a one-second ring-buffer to compute the newest power output (following section *Feature extraction*). The BSI decoder was trained to detect oscillatory activity encoding MI of ankle dorsiflexion, which is expressed as a reduction of this power output in the sensorimotor alpha band. When the cortical activity was decoded as motor imagery, the BSI triggered the ts-MS at 20 Hz, providing contingent feedback. Note that the stimulation could only occur during the periods of motor imagery, to avoid having false positives during the rest periods. The ts-MS pulses activated spinal pools, transmitting efferent volleys to lower-limb muscles, and generated muscular responses every 50 ms (i.e., 20 Hz) (Figure 1B, zooming EMG trace), which were quantified by means of trans-spinal motor evoked potentials (ts-MEP).

To deal with EEG-nonstationarities and potential changes of cortical activation patterns due to ts-MS, we continuously updated the normalization coefficients of the features (initially, the mean and standard deviation of the training dataset). We kept two 48-s buffers, one for rest and one for MI, with the most recent features of these classes. The mean and standard deviation of these two buffers, concatenated together, were used as the normalization coefficients in each iteration before passing the feature vector to the classifier.

#### Median filtering for ts-MS contamination removal

Using electromagnetic currents for stimulating the nervous system can introduce undesirable noise to the neural activity. As characterized in (Insausti-Delgado et al., 2017), the ts-MS distorts the EEG recordings, introducing peaks of short duration (∼10 ms) and large magnitude. A median filter is a suitable method for minimizing the influence of the ts-MS contamination in the EEG signal (Insausti-Delgado et al., 2017, 2021). We applied the median filter as a sliding window of 20 ms in one-sample steps, calculating the median value for each window. This filter attenuates these large peaks preserving the sensorimotor oscillatory activity. A detailed characterization of how this filter can be used to remove similar peaks due to electrical stimulation can be found in (Insausti-Delgado et al., 2021).

### Decoding accuracy

The performance of the classifier for each participant was estimated as the balanced accuracy, calculated as the mean of the true positive rate (TPR) and the true negative rate (TNR) (López-Larraz et al., 2018a). The TPR quantifies the success of the classifier during the MI period, defined as the time interval [1, 4] s. The TNR measures the classifier success during the rest period, which was defined as the time interval [−4, −1] s.

### Neurophysiological measurements

Our BSI has been devised to be used as a rehabilitative tool for patients that have motor impairments. For future interventions based on BSIs, the potential of these systems to interact with cortico-spinal and spino-muscular circuitry is a relevant aspect. With the data recorded during the closed-loop blocks, we conducted some neurophysiological measures to assess the interactions of the BSI with the nervous system.

#### Trans-spinal motor evoked potentials (ts-MEP)

Ts-MS can activate the peripheral nervous system, exciting the spinal nerves at their exit through the intervertebral foramina towards the muscles, resulting in ts-MEPs (Matsumoto et al., 2013). The recruitment of peripheral motor nerves was demonstrated by measuring the ts-MEPs at the tibialis anterior (TA) muscle during spinal stimulation. EMG signals were high-pass filtered at 10 Hz with a 4th order Butterworth filter and trimmed down to 45-ms epochs (from -5–40 ms with respect to the stimulation pulse). Epochs corresponding to the same ts-MS intensity were pooled together and averaged for each subject. The peak-to-peak amplitude of the ts-MEPs for each intensity was determined as the difference between the maximum and the minimum values of the averaged potential.

#### Trans-spinal somatosensory evoked potentials (ts-SEP)

Spinal stimulation can activate the sensory cortex *via* the ascending pathways from the spine, which can be quantified as trans-spinal somatosensory evoked potentials (ts-SEP) (Kunesch et al., 1993). The EEG activity of the CPz channel during closed-loop was high-pass filtered using a 4th order Butterworth filter at 3 Hz. Signals were aligned to the stimulation artifact and epoched to 45-ms periods (from -5–40 ms with respect to the ts-MS pulse). Epochs were grouped according to their intensity and averaged for each subject. The peak-to-peak amplitude of the averaged ts-SEP was calculated as the difference between the maximum and the minimum values for each ts-MS intensity.

### Usability assessments

The participants were asked to evaluate the degree of pain, discomfort and concentration at the end of each closed-loop block. They had to grade between 0 (very low) and 10 (very high): *1)* how painful the stimulation was, *2)* how uncomfortable the stimulation was, and *3)* how easy it was to perform the motor imagery while being stimulated.

### Statistical analysis

We studied the effect of stimulation condition on the BSI performance and on the neurophysiological measurements. The Shapiro-Wilk test was used to determine the Gaussianity of the data. To assess the effect of stimulation on MI decoding accuracy, we used a repeated measures analysis of variance (ANOVA), with stimulation condition as factor (4 levels: ts-MS at 20% of the MSO, ts-MS at 30% of the MSO, ts-MS at 40% of the MSO, and sham stimulation) and decoding accuracy as dependent variable. Post-hoc comparisons were conducted using paired t-tests with Bonferroni correction. To evaluate the influence of the median filter on the BSI performance, we ran paired t-tests with filtering as factor (with and without median filter) and decoding accuracy as dependent variable for each stimulation condition. To study the influence of stimulation intensity on the peak-to-peak amplitude of ts-MEPs and ts-SEPs we used Friedman’s test (3 levels: ts-MS at 20% of the MSO, ts-MS at 30% of the MSO, ts-MS at 40% of the MSO). Paired post-hoc comparisons were performed using the Wilcoxon signed-rank test to analyze significant amplitude differences between intensity pairs. All the statistical tests were conducted in IBM SPSS 25.0 Statistics software (SPSS Inc., Chicago, IL, USA).

## Results

### Control of brain-spine interface using sensorimotor rhythms

Supplementary Movie S1 shows one representative participant controlling the BSI. The participant was asked to rest or to perform MI of the right ankle dorsiflexion, guided by auditory cues. The EEG activity was processed (median filtered, band-pass filtered and OSF filtered) in real-time. To prove the efficacy of the system to remove online stimulation artifacts, the activity of the OSF channel with and without median filtering is displayed. The classifier triggered the ts-MS when the MI brain states were detected. Note that the stimulation was off during resting periods.

### Activating the peripheral and central nervous system by ts-MS

**FIGURE 2:**
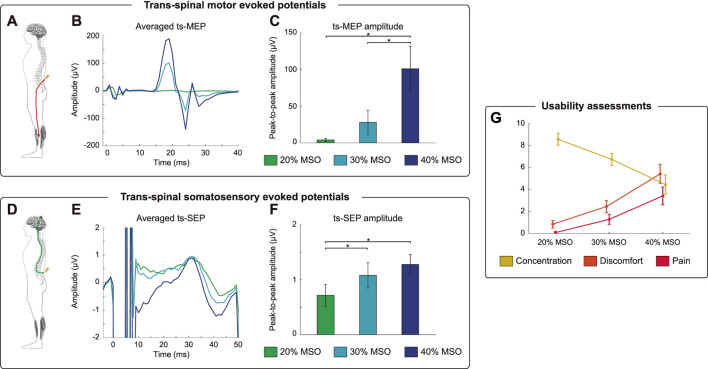
Neurophysiological and usability assessments. **(A)** Recruitment of efferent pathways from the spine to the tibialis anterior (TA) muscle by trans-spinal magnetic stimulation (ts-MS). **(B)** Average of trans-spinal motor evoked potentials (ts-MEPs) of a representative participant for the three different stimulation intensities: 20, 30 and 40% of maximum stimulator output (MSO). **(C)** Mean peak-to-peak amplitude of ts-MEPs and standard errors averaged over all participants for each stimulation intensity. The asterisks indicate significant (*p* < 0.05, Bonferroni corrected) differences in ts-MEP amplitude between ts-MS intensities. **(D)** Ts-MS intensity-dependent recruitment of afferent pathways from the spine to the cortex *via* ts-MS as measured by trans-spinal somatosensory evoked potentials (ts-SEP). **(E)** Average ts-SEPs of a representative participant for the three different stimulation intensities. Notice that averages ts-MEPs **(B)** and averaged ts-SEPs **(E)** have different scales. **(F)** Mean peak-to-peak amplitude of ts-SEPs and standard errors averaged over all participants for each stimulation intensity. The asterisks indicate significant (*p* < 0.05, Bonferroni corrected) differences in ts-SEP amplitude between ts-MS intensities. **(G)** Usability scores for concentration, discomfort and pain, averaged over all participants.

The BSI has been engineered for neurorehabilitative purposes, on the premise that it could exploit Hebbian mechanisms and facilitate motor recovery in patients with motor impairment. To elucidate to what extent the closed-loop ts-MS can interact with the peripheral and the central nervous system, we conducted some neurophysiological measurements. By analyzing the ts-MEPs, we assessed the neuromodulatory effects of the stimulation on the peripheral nervous system (Figure 2A). We extracted the ts-MEPs from the closed-loop stimulation blocks (Figure 2B) and compared their amplitude according to the stimulation intensity. The peak-to-peak amplitude of the ts-MEPs was significantly affected by the intensity used (Friedman’s χ2(2) = 15.80; *p* < 0.001). Post-hoc comparisons revealed significantly larger ts-MEPs amplitudes at 40% of the MSO compared to 20% (Z = −2.803, *p* = 0.015) and compared to 30% (Z = -2.803, *p* = 0.015) (Figure 2C).

To assess the neuromodulatory effects of the stimulation on the central nervous system, we computed the trans-spinal somatosensory evoked potentials (ts-SEPs) (Figure 2D). As for the ts-MEPs, we averaged the ts-SEPs for each subject, grouping by stimulation intensity. The ts-MS produced a positive peak with a latency of 30 ms and a negative peak at 40 ms (Figure 2E), according to the conduction times presented in previous literature (Kunesch et al., 1993). The peak-to-peak amplitude of the ts-SEP was significantly affected by the stimulation intensity (Friedman’s χ2(2) = 9.80; *p* = 0.007). Post-hoc paired comparisons showed significantly smaller ts-SEPs when stimulating at 20% compared to 30% (Z = −2.599, *p* = 0.027) or 40% (Z = −2.701, *p* = 0.021) of the MSO (Figure 2F).

### Usability and perception of the users

Our descriptive analysis on usability shows that the participants perceived more discomfort and pain, and decreased concentration on the MI task, as the intensity of ts-MS increased (Figure 2G). All the participants described the stimulation at higher intensities as uncomfortable, rather than painful. No adverse effects due to ts-MS were reported by, or observed in, any of the participants.

### Feature extraction and online decoding accuracy

Before using the closed-loop system, the participants underwent a short screening phase, where neural signatures of the sensorimotor alpha rhythm encoding motor intentions were identified to build a classifier. Seven out of the ten participants showed strong cortical activation patterns during MI in the screening data, revealed as a significant event-related desynchronization (ERD) in alpha and beta bands (Supplementary Figure S1).

The average decoding accuracies for all the participants were 61.7%, 57.9%, 58.4% and 59.6% for sham stimulation, ts-MS at 20% of the MSO, ts-MS at 30% of the MSO and ts-MS at 40% of the MSO, respectively (Supplementary Figure S2A). There was no significant effect of stimulation condition on decoding accuracy, as revealed by the repeated measures ANOVA (F(3, 27) = 1.912, *p* = 0.151). As a post-hoc analysis, we discarded the data of the three participants who did not show a modulation of the sensorimotor alpha rhythm during MI in the screening phase (subjects 3, 5 and 8 in Supplementary Figure S1). These three participants had a decoding accuracy around chance level in the closed-loop phase for every stimulation condition. When we excluded them from the analysis, the average decoding accuracy increased up to 66.2%, 61.8%, 63.5% and 66.1%, respectively (Supplementary Figure S2B). These values were not significantly different between ts-MS intensities either (repeated measures ANOVA, F(3, 18) = 2.008, *p* = 0.149).

### Removing stimulation artifacts from the EEG

**FIGURE 3:**
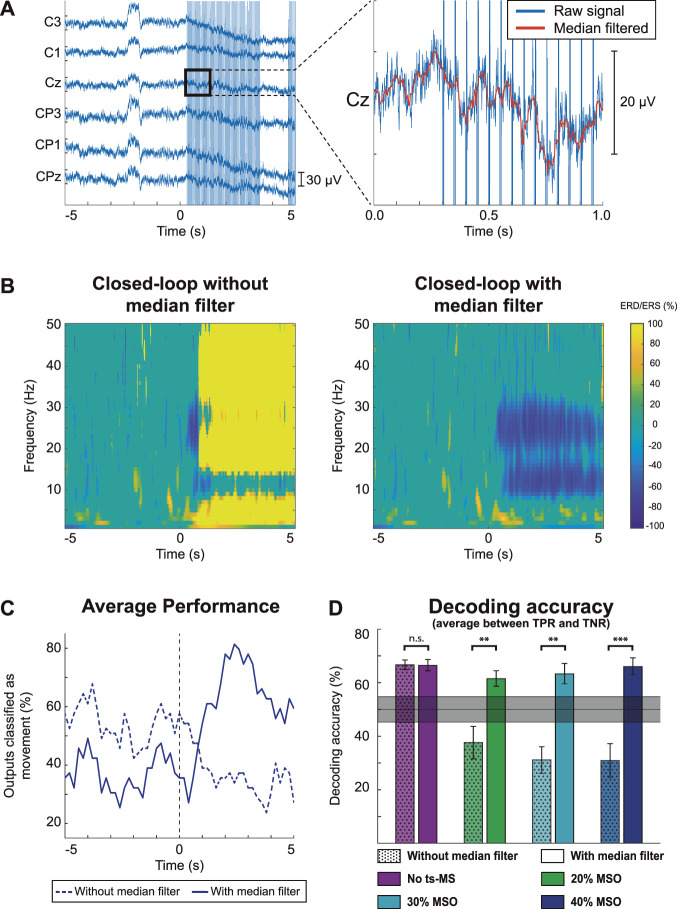
Characterization of stimulation artifacts and their effects on cortical activity and decoding accuracy. The results for one representative participant are displayed in the most unfavorable scenario, with stimulation at 40% of the maximum stimulator output (MSO). **(A)** EEG trace of one trial, showing the effect of the stimulation as high-amplitude artifacts. Zooming into a one-second segment, the details of the signal without (blue) or with (orange) median filtering can be appreciated. **(B)** Grand average time-frequency maps without (left) and with (right) median filter. Time 0 s corresponds to the display of the auditory cue to start the motor imagery (MI). **(C)** Average time-response of the classifier without (dashed) and with (solid) median filter. **(D)** Decoding accuracy calculated as the mean between true negative rate (TNR) and true positive rate (TPR) for each stimulation condition with and without median filter for the seven participants with detectable MI-related desynchronization in the alpha frequency band. The shaded gray area shows the confidence interval of the chance level (alpha = 0.05), calculated based on all the test trials, according to (Müller-Putz et al., 2008). The asterisks indicate significant differences (** for *p* < 0.01, *** for *p* < 0.001) between decoding accuracies when median filtering is applied.

We also studied how ts-MS affects the cortical activity and the performance of the BSI. Stimulation artifacts distort the ongoing EEG activity, hindering its processing. The median filter effectively eliminated the high-amplitude peaks, allowing the quantification of sensorimotor modulation (Figure 3A). Figure 3B displays the estimated cortical activity of a representative participant during closed-loop ts-MS at 40% of the MSO (i.e., the highest stimulation intensity) with and without applying the median filtering. If the stimulation artifacts are not eliminated, they are observable in the EEG as a broadband event-related synchronization (ERS), or power increase, covering the frequencies of interest (Figure 3B left). Median filtering revealed the significant event-related desynchronization (ERD) of alpha and beta frequencies (Figure 3B right), which allowed the classifier to decode the MI (Figure 3C). We calculated the decoding accuracy of MI with and without median filter for each stimulation condition for those participants with detectable ERD. Paired t-tests revealed that applying the median filter leaded to significantly higher decoding accuracies in closed-loop ts-MS at 20% (*t*(6) = −3.821, *p* = 0.009), ts-MS at 30% (*t*(6) = −5.215, *p* = 0.002) and ts-MS at 40% of the MSO (*t*(6) = −6.555, *p* = 0.001) (Figure 3D).

## Discussion

In this paper, we report on the first non-invasive brain-spine interface (BSI), based on the continuous control of trans-spinal magnetic stimulation (ts-MS) guided by EEG. Our BSI enables the direct association of cortical activity encoding motor intentions with the activation of afferent (from spine to somatosensory cortex) and efferent (from spine to lower-limb muscles) pathways. This natural approach to link brain activity with the peripheral nervous system, as during walking, could be used to neuromodulate sensory-motor excitability to exploit neuroplasticity and constitute a relevant tool for rehabilitation of paralyzed patients. Although in this study we did not measure the long-term neuromodulatory or therapeutic effects, we validated feasibility, safety and usability of the BSI, which provides sufficient basis towards designing and evaluating interventions based on this innovative approach.

Brain-controlled spinal cord stimulation can potentially be employed for assistive or rehabilitative purposes by patients with lower-limb paralysis. Spinal cord stimulation has been used to neuromodulate the spinal circuitry, supporting motor recovery after lower-limb paralysis (Edgerton and Roy, 2012; Megía García et al., 2019). The first studies in animals evidenced the neuromodulatory properties of spinal dorsal root stimulation (Budakova, 1972; Grillner and Zangger, 1979). Later investigations using epidural spinal stimulation demonstrated the capacity of stimulation to facilitate locomotor-like patterns and to produce long-lasting motor recovery after intensive training in paralyzed animals (Ichiyama et al., 2005; Gerasimenko et al., 2008; Wenger et al., 2016) and SCI patients (Grahn et al., 2017; Gill et al., 2018). However, controlling and modulating the stimulation based on brain activation is a more natural approach than continuously stimulating the spinal circuits (McPherson et al., 2015). In fact, the contingent association of cortical activity produced by the intention to move a paralyzed limb and the afferent volley generated by spinal stimulation can exploit Hebbian mechanisms and facilitate functional recovery (Ramos-Murguialday et al., 2013; Mrachacz-Kersting et al., 2016; Bonizzato et al., 2018). This association is the basic operating principle of brain-spine interfaces (Alam et al., 2016).

Brain-spine interfaces developed to date involve implantable technologies and have only been tested in animal models (Nishimura et al., 2013b; Alam et al., 2014; McPherson et al., 2015; Capogrosso et al., 2016; Bonizzato et al., 2018). Compared with continuous spinal stimulation, brain-controlled stimulation has been shown to enhance stepping quality and accelerate locomotor recovery (Capogrosso et al., 2016; Bonizzato et al., 2018). Developing a non-invasive BSI would facilitate experimental testing with healthy and paralyzed subjects and would allow us to compare the effectiveness of Hebbian-based closed-loop stimulation with passive stimulation, as previously done in animals. In humans, the only approaches presenting closed-loop volitional control of spinal stimulation proposed non-brain-commanded paradigms. On one hand, Nishimura and colleagues proposed the use of arm EMG to control non-invasive magnetic spinal stimulation in healthy subjects and SCI patients (Sasada et al., 2014; Nakao et al., 2015). On the other hand, Courtine and colleagues implanted epidural electrical stimulation electrodes in the lumbar spinal cord of SCI patients and used inertial measurement units (IMUs) located on the feet to control the stimulation (Wagner et al., 2018). Our approach relied on extracting motor commands non-invasively from brain activity by EEG to provide closed-loop control of ts-MS. This associative link between cortical activation due the motor intention and the efferent/afferent activation of spinal pathways primes the sensory-motor network involved in gait and favors neuroplasticity and Hebbian mechanisms, which could be boosted by adjuvant training (Seáñez and Capogrosso, 2021), such as behaviorally-oriented physiotherapy, to promote functional motor recovery.

Non-invasive brain-machine interfaces (BMIs) allow the transmission of volitional cortical commands to control rehabilitative devices (Wolpaw et al., 2002; López-Larraz et al., 2018b; Guggenberger et al., 2020). For instance, there is ample evidence demonstrating contingent EEG control of robotic exoskeletons with patients (Ramos-Murguialday et al., 2013; López-Larraz et al., 2018a). Electric and magnetic neurostimulation can also be integrated in non-invasive BMIs. However, to date, contingent online control of such neurostimulators has not been achieved, since the stimulation introduces artifacts that hinder extracting reliable information from the brain activity recordings. Therefore, BMIs integrating neurostimulation have only been proposed triggering predefined stimulation patterns, not allowing continuous control nor contingency (Pfurtscheller et al., 2005; Trincado-Alonso et al., 2017; Biasiucci et al., 2018).

Dealing with stimulation artifacts is a challenge for closed-loop neural interfaces. Different approaches have been proposed for cleaning stimulation contamination from neural recordings, such as blanking, interpolation or linear regression reference (Walter et al., 2012; Young et al., 2018). For invasive recordings, regression methods have been proven effective to eliminate stimulation artifacts (Young et al., 2018), mainly due to the low inter-electrode impedance variability and within-session stability, allowing closed-loop neurostimulation (Bouton et al., 2016; Ajiboye et al., 2017). However, none of these methods has been proven effective for EEG recordings, and the estimation of cortical activation during stimulation is biased even if blanking or interpolation of the artifacts is used (Walter et al., 2012).

We proposed the median filter, since it can eliminate high-amplitude peaks in a time-series without causing signal discontinuities (which is the main problem of blanking or interpolation). Its main limitation is that it attenuates the activity at higher frequencies (exponential attenuation between 0 Hz and the 1/*W* Hz, with *Ws* being the length of the window of the median filter) (Insausti-Delgado et al., 2021). However, this frequency-dependent attenuation does not have a big impact on the sensorimotor alpha oscillations (7–15 Hz) that we used to detect the motor imagery. To the best of our knowledge, this is the first time a non-invasive closed-loop system controls the stimulation in real-time and effectively deals with these artifacts.

Our adaptive decoder successfully dealt with the brain activity changes due to ts-MS. The decoding accuracy was within the level of acceptance for closed-loop rehabilitative neuroprosthetics (Ramos-Murguialday et al., 2013). The control condition of sham stimulation allowed us to assess if the closed-loop performance was similar (i.e., not significantly different) between the stimulation conditions (i.e., ts-MS at 20%, ts-MS at 30% and ts-MS at 40%) and no stimulation. Remarkably, we demonstrated that the accuracy was not influenced by the stimulation intensity, proving the robustness and efficiency of the proposed methodology for artifact removal, which in turn allows the implementation of different stimulation protocols, such as above- or below-motor threshold (note that the spinal motor threshold of healthy subjects is usually between 25% and 40% of the MSO). We assume that the conclusions drawn from our results could also apply to other stimulation approaches that do not target the spinal cord, such as functional electrical stimulation, neuromuscular electrical stimulation, or transcranial magnetic stimulation, since the stimulation would spread through the human body to the recording electrodes, contaminating the brain signals and biasing the EEG decoder. All participants reported a decreased usability of the system with higher stimulation intensities (i.e., more pain and discomfort, and less concentration on the task). However, their subjective usability perception at high intensity did not affect the performance of the BSI.

Previous studies evidenced that combining MI of ankle dorsiflexion with peripheral stimulation increases corticomuscular efficacy in humans (Kaneko et al., 2014; Takahashi et al., 2019; Yasui et al., 2019). Thus, we believe that our BSI-based training could also upregulate the excitability of the entire sensory-motor network involved in gait and, in turn, could be used as a tool to prime behaviorally-oriented physiotherapy or robot-assisted therapy (Asín-Prieto et al., 2020) by improving clinical benefits of neurorehabilitative interventions. We hypothesize that once lower limb sensorimotor brain activation is detected, top-down commands are being sent through the spine to the muscles, and that a SCS at the nerve root corresponding the motoneurons should facilitate the recruitment of more fibers delivering the top-down command to the muscles resulting in higher corticomuscular synaptic efficacy. Furthermore, the feedback (stimulation is perceived through receptors in the skin and muscles on the stimulation area but also through the muscle contractions and the antidromic stimulation afferent evoked response) will induce an operant conditioning and instrumental learning effect exciting the comprising neural network (brain-spine-muscles). The next natural step following this study would be to demonstrate the efficacy of the brain-controlled stimulation to induce neuroplasticity in human nervous system supporting locomotor functional recovery, as it has already been proven invasively in rodents and primates (McPherson et al., 2015; Capogrosso et al., 2016, 2018; Bonizzato et al., 2018). An exhaustive battery of assessments should be conducted, evaluating motor and sensory pathways and spinal neural processes, to precisely characterize the neurophysiological effects of a BSI-based intervention. The involvement of ipsilateral and contralateral muscles while being stimulated during a MI task is also a key aspect that should be investigated and could determine training-induced neuroplastic adaptations. Although we did not conduct synaptic efficacy assessments in this preliminary analysis, we studied the neuromodulatory effects during closed-loop stimulation. We demonstrated the capability of our platform to engage the central and peripheral nervous systems as expected. The ts-MS activated efferent pathways, inducing ts-MEPs in lower-limb muscles, and afferent pathways, producing ts-SEPs. These findings prove that both afferent and efferent neuromodulation are intensity dependent, confirming previous results (Matsumoto et al., 2013; Rossini et al., 2015). This fact implies that we could upregulate motor excitability at spinal and peripheral level simultaneously with sensory processing at cortical level, in a similar way these neural networks are activated during natural gait. Most importantly, this timely-linked efferent and afferent neural activations contribute to the integration of motor task preparation/execution with sensory feedback to the cortex, which is key in motor learning and cortical reorganization after the injury (Mohammed and Hollis, 2018).

In conclusion, in this study we have proposed and validated the first non-invasive BSI. Further research should focus on studying the feasibility of this system as a rehabilitative tool in paralyzed patients. Future interventional protocols should also consider activity-based rehabilitation including, for instance, standing or stepping on a treadmill with weight support, and investigate the associated motion artifacts that would conceivably impair brain correlates detection and, in turn, decoding accuracy. The role of ts-MS parameters (i.e., frequency, intensity, dose, etc.) on the excitability of spinal neural networks should also be disclosed. Computational modelling might be a key tool to understand the ongoing mechanisms involved in spinal neuromodulation due to ts-MS and to optimize interventions based on spinal stimulation for functional recovery (Formento et al., 2018; Khadka et al., 2020; Greiner et al., 2021). Recent approaches for EEG decoding, based on advanced machine learning algorithms, have received substantial attention and could also be implemented for designing and training classifiers that could considerably boost the performance of closed-loop systems (Schirrmeister et al., 2017; Saeidi et al., 2021). Nevertheless, the here presented findings constitute the first steps towards the application of non-invasive BSIs as a novel neuroscientific and therapeutic tool.

## Data Availability

The raw data supporting the conclusion of this article will be made available by the authors, without undue reservation.

## References

[B1] Ajiboye A. B. Willett F. R. Young D. R. Memberg W. D. Murphy B. A. Miller J. P. (2017). Restoration of reaching and grasping movements through brain-controlled muscle stimulation in a person with tetraplegia: A proof-of-concept demonstration. Lancet 389, 1821–1830. 10.1016/S0140-6736(17)30601-3 28363483 PMC5516547

[B2] Alam M. Chen X. Zhang Z. Li Y. He J. (2014). A brain-machine-muscle interface for restoring hindlimb locomotion after complete spinal transection in rats. PLoS ONE 9, e103764. 10.1371/journal.pone.0103764 25084446 PMC4118921

[B3] Alam M. Rodrigues W. Pham B. N. Thakor N. v. (2016). Brain-machine interface facilitated neurorehabilitation via spinal stimulation after spinal cord injury: Recent progress and future perspectives. Brain Res. 1646, 25–33. 10.1016/j.brainres.2016.05.039 27216571

[B4] Alam M. Garcia-alias G. Jin B. Keyes J. Zhong H. Roy R. R. (2017). Electrical neuromodulation of the cervical spinal cord facilitates forelimb skilled function recovery in spinal cord injured rats. Exp. Neurol. 291, 141–150. 10.1016/j.expneurol.2017.02.006 28192079 PMC6219872

[B5] Asín-Prieto G. Martínez-Expósito A. Barroso F. O. Urendes E. J. Gonzalez-Vargas J. Alnajjar F. S. (2020). Haptic adaptive feedback to promote motor learning with a robotic ankle exoskeleton integrated with a video game. Front. Bioeng. Biotechnol. 8, 113. 10.3389/fbioe.2020.00113 32154239 PMC7047324

[B6] Beaulieu L. D. Schneider C. (2013). Effects of repetitive peripheral magnetic stimulation on normal or impaired motor control. A review. Neurophysiol. Clinique/Clinical Neurophysiol. 43, 251–260. 10.1016/j.neucli.2013.05.003 24094911

[B7] Biasiucci A. Leeb R. Iturrate I. Perdikis S. Al-Khodairy A. Corbet T. (2018). Brain-actuated functional electrical stimulation elicits lasting arm motor recovery after stroke. Nat. Commun. 9, 2421. 10.1038/s41467-018-04673-z 29925890 PMC6010454

[B8] Bonizzato M. Pidpruzhnykova G. DiGiovanna J. Shkorbatova P. Pavlova N. Micera S. (2018). Brain-controlled modulation of spinal circuits improves recovery from spinal cord injury. Nat. Commun. 9, 1–14. 10.1038/s41467-018-05282-6 30068906 PMC6070513

[B9] Bouton C. E. Shaikhouni A. Annetta N. v. Bockbrader M. A. Friedenberg D. A. Nielson D. M. (2016). Restoring cortical control of functional movement in a human with quadriplegia. Nature 533, 247–250. 10.1038/nature17435 27074513

[B10] Budakova N. N. (1972). Stepping movements evoked by repetitive dorsal root stimulation in a mesencephalic cat. Neurosci. Behav. Physiol. 5, 355–363. 10.1007/BF01183110 4210196

[B11] Capogrosso M. Milekovic T. Borton D. Wagner F. Moraud E. M. Mignardot J. B. (2016). A brain-spine interface alleviating gait deficits after spinal cord injury in primates. Nature 539, 284–288. 10.1038/nature20118 27830790 PMC5108412

[B12] Capogrosso M. Wagner F. B. Gandar J. Moraud E. M. Wenger N. Milekovic T. (2018). Configuration of electrical spinal cord stimulation through real-time processing of gait kinematics. Nat. Protoc. 13, 2031–2061. 10.1038/s41596-018-0030-9 30190556

[B13] Edgerton V. R. Roy R. R. (2012). A new age for rehabilitation. Eur. J. Phys. Rehabil. Med. 48, 99–109. 22407010

[B14] Formento E. Minassian K. Wagner F. Mignardot J. B. le Goff-Mignardot C. G. Rowald A. (2018). Electrical spinal cord stimulation must preserve proprioception to enable locomotion in humans with spinal cord injury. Nat. Neurosci. 21, 1728–1741. 10.1038/s41593-018-0262-6 30382196 PMC6268129

[B15] Gerasimenko Y. Roy R. R. Edgerton V. R. (2008). Epidural stimulation: Comparison of the spinal circuits that generate and control locomotion in rats, cats and humans. Exp. Neurol. 209, 417–425. 10.1016/j.expneurol.2007.07.015 17850791 PMC2288525

[B16] Gerasimenko Y. Gorodnichev R. Machueva E. Pivovarova E. Semyenov D. Savochin A. (2010). Novel and direct access to the human locomotor spinal circuitry. J. Neurosci. 30, 3700–3708. 10.1523/JNEUROSCI.4751-09.2010 20220003 PMC2847395

[B17] Gill M. L. Grahn P. J. Calvert J. S. Linde M. B. Lavrov I. A. Strommen J. A. (2018). Neuromodulation of lumbosacral spinal networks enables independent stepping after complete paraplegia. Nat. Med. 24, 1677–1682. 10.1038/s41591-018-0175-7 30250140

[B18] Grahn P. J. Lavrov I. A. Sayenko D. G. Straaten M. G. Van Gill M. L. Strommen J. A. (2017). Enabling task-specific volitional motor functions via spinal cord neuromodulation in a human with paraplegia. Mayo Clin. Proc. 92, 544–554. 10.1016/j.mayocp.2017.02.014 28385196

[B19] Graimann B. Pfurtscheller G. (2006). Quantification and visualization of event-related changes in oscillatory brain activity in the time – frequency domain. Prog. Brain Res. 159, 79–97. 10.1016/S0079-6123(06)59006-5 17071225

[B20] Greiner N. Barra B. Schiavone G. Lorach H. James N. Conti S. (2021). Recruitment of upper-limb motoneurons with epidural electrical stimulation of the cervical spinal cord. Nat. Commun. 12, 435. 10.1038/s41467-020-20703-1 33469022 PMC7815834

[B21] Grillner S. Zangger P. (1979). On the central generation of locomotion in the low spinal cat. Exp. Brain Res. 34, 241–261. 10.1007/BF00235671 421750

[B22] Guggenberger R. Heringhaus M. Gharabaghi A. (2020). Brain-machine neurofeedback: Robotics or electrical stimulation? Front. Bioeng. Biotechnol. 8, 639. 10.3389/fbioe.2020.00639 32733860 PMC7358603

[B23] Harkema S. Gerasimenko Y. Hodes J. Burdick J. Angeli C. Chen Y. (2011). Effect of epidural stimulation of the lumbosacral spinal cord on voluntary movement, standing, and assisted stepping after motor complete paraplegia: A case study. Lancet 377, 1938–1947. 10.1016/S0140-6736(11)60547-3 21601270 PMC3154251

[B25] Ichiyama R. M. Gerasimenko Y. P. Zhong H. Roy R. R. Edgerton V. R. (2005). Hindlimb stepping movements in complete spinal rats induced by epidural spinal cord stimulation. Neurosci. Lett. 383, 339–344. 10.1016/j.neulet.2005.04.049 15878636

[B26] Insausti-Delgado A. López-Larraz E. Bibián C. Nishimura Y. Birbaumer N. Ramos-Murguialday A. (2017). “Influence of trans-spinal magnetic stimulation in electrophysiological recordings for closed-loop rehabilitative systems,” in Proceedings of the 39th Annual International Conference of the IEEE Engineering in Medicine and Biology Society (EMBC), 2518–2521. 10.1109/EMBC.2017.8037369 29060411

[B27] Insausti-Delgado A. Lόpez-Larraz E. Birbaumer N. Ramos-Murguialday A. (2018). “An exhaustive assessment to quantify changes in spinal excitability after spinal-cord stimulation,” in 11th FENS Forum of Neuroscience.

[B28] Insausti-Delgado A. López-Larraz E. Nishimura Y. Birbaumer N. Ziemann U. Ramos-Murguialday A. (2019). “Quantifying the effect of trans-spinal magnetic stimulation on spinal excitability,” in Proceeding of the 9th International IEEE EMBS Conference on Neural Engineering (NER), San Francisco, CA, USA, March 2019 (IEEE). 10.1109/NER.2019.8717016

[B29] Insausti-Delgado A. López-Larraz E. Nishimura Y. Ziemann U. Ramos-Murguialday A. (2020). Non-invasive brain-spine interface: Continuous brain control of trans-spinal magnetic stimulation using EEG. bioRxiv. 10.1101/2020.08.10.230912 PMC9659618 36394044

[B30] Insausti-Delgado A. López-Larraz E. Omedes J. Ramos-Murguialday A. (2021). Intensity and dose of neuromuscular electrical stimulation influence sensorimotor cortical excitability. Front. Neurosci. 14, 593360. 10.3389/fnins.2020.593360 33519355 PMC7845652

[B31] Jackson A. Zimmermann J. B. (2012). Neural interfaces for the brain and spinal cord—Restoring motor function. Nat. Rev. Neurol. 8, 690–699. 10.1038/nrneurol.2012.219 23147846

[B32] Kaneko F. Hayami T. Aoyama T. Kizuka T. (2014). Motor imagery and electrical stimulation reproduce corticospinal excitability at levels similar to voluntary muscle contraction. J. NeuroEngineering Rehabilitation 11, 94–97. 10.1186/1743-0003-11-94 PMC4113028 24902891

[B33] Kato K. Sawada M. Nishimura Y. (2019). Bypassing stroke-damaged neural pathways via a neural interface induces targeted cortical adaptation. Nat. Commun. 10, 4699. 10.1038/s41467-019-12647-y 31619680 PMC6796004

[B34] Khadka N. Liu X. Zander H. Swami J. Rogers E. Lempka S. (2020). Realistic anatomically detailed open-source spinal cord stimulation (RADO-SCS) model. J. Neural Eng. 17, 026033. 10.1088/1741-2552/ab8344 32209741

[B35] Knikou M. (2013). Neurophysiological characteristics of human leg muscle action potentials evoked by transcutaneous magnetic stimulation of the spine. Bioelectromagnetics 34, 200–210. 10.1002/bem.21768 23192827

[B36] Krause P. Edrich T. Straube A. (2004). Lumbar repetitive magnetic stimulation reduces spastic tone increase of the lower limbs. Spinal Cord. 42, 67–72. 10.1038/sj.sc.3101564 14765138

[B37] Kunesch E. Knecht S. Classen J. Roick H. Tyercha C. Benecke R. (1993). Somatosensory evoked potentials (SEPs) elicited by magnetic nerve stimulation. Electroencephalogr. Clin. Neurophysiology/Evoked Potentials Sect. 88, 459–467. 10.1016/0168-5597(93)90035-N 7694832

[B38] López-Larraz E. Montesano L. Gil-Agudo Á. Minguez J. (2014). Continuous decoding of movement intention of upper limb self-initiated analytic movements from pre-movement EEG correlates. J. Neuroeng. Rehabil. 11, 153. 10.1186/1743-0003-11-153 25398273 PMC4247645

[B39] López-Larraz E. Montesano L. Gil-Agudo Á. Minguez J. Oliviero A. (2015). Evolution of EEG motor rhythms after spinal cord injury: A longitudinal study. PLoS ONE 10, e0131759. 10.1371/journal.pone.0131759 26177457 PMC4503564

[B40] López-Larraz E. Figueiredo T. C. Insausti-Delgado A. Ziemann U. Birbaumer N. Ramos-Murguialday A. (2018a). Event-related desynchronization during movement attempt and execution in severely paralyzed stroke patients: An artifact removal relevance analysis. NeuroImage Clin. 20, 972–986. 10.1016/j.nicl.2018.09.035 30312940 PMC6180341

[B41] López-Larraz E. Sarasola-Sanz A. Irastorza-Landa N. Birbaumer N. Ramos-Murguialday A. (2018b). Brain-machine interfaces for rehabilitation in stroke: A review. NeuroRehabilitation 43, 77–97. 10.3233/NRE-172394 30056435

[B42] Matsumoto H. Hanajima R. Terao Y. Ugawa Y. (2013). Magnetic-motor-root stimulation: Review. Clin. Neurophysiol. 124, 1055–1067. 10.1016/j.clinph.2012.12.049 23485367

[B43] McPherson J. G. Miller R. R. Perlmutter S. I. McPherson J. G. Robert R. Miller S. I. P. (2015). Targeted, activity-dependent spinal stimulation produces long-lasting motor recovery in chronic cervical spinal cord injury. Proc. Natl. Acad. Sci. U. S. A. 78, 12193–12198. 10.1073/pnas.1505383112 PMC4593107 26371306

[B44] Megía García A. Serrano-Muñoz D. Taylor J. Avendaño-Coy J. Gómez-Soriano J. (2019). Transcutaneous spinal cord stimulation and motor rehabilitation in spinal cord injury: A systematic review. Neurorehabil. Neural Repair 34, 3–12. 10.1177/1545968319893298 31858871

[B46] Mohammed H. Hollis E. R. (2018). Cortical reorganization of sensorimotor systems and the role of intracortical circuits after spinal cord injury. Neurotherapeutics 15, 588–603. 10.1007/s13311-018-0638-z 29882081 PMC6095783

[B47] Mrachacz-Kersting N. Jiang N. Stevenson A. J. T. Niazi I. K. Kostic V. Pavlovic A. (2016). Efficient neuroplasticity induction in chronic stroke patients by an associative brain-computer interface. J. Neurophysiol. 115, 1410–1421. 10.1152/jn.00918.2015 26719088 PMC4808132

[B48] Müller-Putz G. Scherer R. Brunner C. Leeb R. Pfurtscheller G. (2008). Better than random: A closer look on BCI results. Int. J. Bioelectromagn. 10, 52–55.

[B49] Nakao Y. Sasada S. Kato K. Murayama T. Kadowaki S. S Y. (2015). “Restoring walking ability in individuals with severe spinal cord injury using a closed-loop spinal magnetic stimulation,” in Proceedings of the 45th annual meeting of society for neuroscience. Available at: https://www.abstractsonline.com/Plan/ViewAbstract.aspx?sKey=70d620e9-53bf-4bef-858d-e79452be5b51&cKey=5f31c5f3-9c0c-4873-8cd1-2fab3b718c7d&mKey=d0ff4555-8574-4fbb-b9d4-04eec8ba0c84 .

[B50] Nardone R. Höller Y. Taylor A. Thomschewski A. Orioli A. Frey V. (2015). Noninvasive spinal cord stimulation: Technical aspects and therapeutic applications. Neuromodulation Technol. A. T. Neural Interface 18, 580–591. 10.1111/ner.12332 26245458

[B51] Naros G. Naros I. Grimm F. Ziemann U. Gharabaghi A. (2016). Reinforcement learning of self-regulated sensorimotor β-oscillations improves motor performance. Neuroimage 134, 142–152. 10.1016/j.neuroimage.2016.03.016 27046109

[B52] Naros G. Lehnertz T. Leão M. T. Ziemann U. Gharabaghi A. (2020). Brain state-dependent gain modulation of corticospinal output in the active motor system. Cereb. Cortex 30, 371–381. 10.1093/cercor/bhz093 31204431

[B53] Neuper C. Scherer R. Reiner M. Pfurtscheller G. (2005). Imagery of motor actions: Differential effects of kinesthetic and visual-motor mode of imagery in single-trial EEG. Cognitive Brain Res. 25, 668–677. 10.1016/j.cogbrainres.2005.08.014 16236487

[B54] Nishimura Y. Perlmutter S. I. Eaton R. W. Fetz E. E. (2013a). Spike-timing-dependent plasticity in primate corticospinal connections induced during free behavior. Neuron 80, 1301–1309. 10.1016/j.neuron.2013.08.028 24210907 PMC4079851

[B55] Nishimura Y. Perlmutter S. I. Fetz E. E. (2013b). Restoration of upper limb movement via artificial corticospinal and musculospinal connections in a monkey with spinal cord injury. Front. Neural Circuits 7, 57. 10.3389/fncir.2013.00057 23596396 PMC3622884

[B56] Pfurtscheller G. Müller-Putz G. R. Pfurtscheller J. Rupp R. (2005). EEG-based asynchronous BCI controls functional electrical stimulation in a tetraplegic patient. EURASIP J. Adv. Signal Process. 2005, 3152–3155. 10.1155/ASP.2005.3152

[B57] Powell M. P. Verma N. Sorensen E. Carranza E. Boos A. Fields D. (2022). Epidural stimulation of the cervical spinal cord improves voluntary motor control in post-stroke upper limb paresis. medRxiv. 10.1101/2022.04.11.22273635 PMC10291889 36807682

[B58] Ramos-Murguialday A. Birbaumer N. (2015). Brain oscillatory signatures of motor tasks. J. Neurophysiol. 113, 3663–3682. 10.1152/jn.00467.2013 25810484 PMC4468978

[B59] Ramos-Murguialday A. Schürholz M. Caggiano V. Wildgruber M. Caria A. Hammer E. M. (2012). Proprioceptive feedback and brain computer interface (BCI) based neuroprostheses. PLoS ONE 7, e47048. 10.1371/journal.pone.0047048 23071707 PMC3465309

[B60] Ramos-Murguialday A. Broetz D. Rea M. Läer L. Yilmaz Ö. Brasil F. L. (2013). Brain-machine interface in chronic stroke rehabilitation: A controlled study. Ann. Neurol. 74, 100–108. 10.1002/ana.23879 23494615 PMC3700597

[B61] Rossini P. M. Burke D. Chen R. Cohen L. G. Daskalakis Z. Di Iorio R. (2015). Non-invasive electrical and magnetic stimulation of the brain, spinal cord, roots and peripheral nerves: Basic principles and procedures for routine clinical and research application. An updated report from an I.F.C.N. Committee. Clin. Neurophysiol. 126, 1071–1107. 10.1016/j.clinph.2015.02.001 25797650 PMC6350257

[B62] Saeidi M. Karwowski W. Farahani F. v. Fiok K. Taiar R. Hancock P. A. (2021). Neural decoding of EEG signals with machine learning: A systematic review. Brain Sci. 11, 1525. 10.3390/brainsci11111525 34827524 PMC8615531

[B63] Sasada S. Kato K. Kadowaki S. Groiss S. J. Ugawa Y. Komiyama T. (2014). Volitional walking via upper limb muscle-controlled stimulation of the lumbar locomotor center in man. J. Neurosci. 34, 11131–11142. 10.1523/JNEUROSCI.4674-13.2014 25122909 PMC6705266

[B65] Schirrmeister R. T. Springenberg J. T. Fiederer L. D. J. Glasstetter M. Eggensperger K. Tangermann M. (2017). Deep learning with convolutional neural networks for EEG decoding and visualization. Hum. Brain Mapp. 38, 5391–5420. 10.1002/hbm.23730 28782865 PMC5655781

[B66] Seáñez I. Capogrosso M. (2021). Motor improvements enabled by spinal cord stimulation combined with physical training after spinal cord injury: Review of experimental evidence in animals and humans. Bioelectron. Med. 7, 16. 10.1186/s42234-021-00077-5 34706778 PMC8555080

[B67] Seeck M. Koessler L. Bast T. Leijten F. Michel C. Baumgartner C. (2017). The standardized EEG electrode array of the IFCN. Clin. Neurophysiol. 128, 2070–2077. 10.1016/j.clinph.2017.06.254 28778476

[B68] Takahashi Y. Kawakami M. Yamaguchi T. Idogawa Y. Tanabe S. Kondo K. (2019). Effects of leg motor imagery combined with electrical stimulation on plasticity of corticospinal excitability and spinal reciprocal inhibition. Front. Neurosci. 13, 149. 10.3389/fnins.2019.00149 30846928 PMC6393385

[B70] Trincado-Alonso F. López-Larraz E. Resquín F. Ardanza A. Pérez-Nombela S. Pons J. L. (2017). A pilot study of brain-triggered electrical stimulation with visual feedback in patients with incomplete spinal cord injury. J. Med. Biol. Eng. 38, 790–803. 10.1007/s40846-017-0343-0

[B71] Wagner F. B. Mignardot J. B. le Goff-Mignardot C. G. Demesmaeker R. Komi S. Capogrosso M. (2018). Targeted neurotechnology restores walking in humans with spinal cord injury. Nature 563, 65–71. 10.1038/s41586-018-0649-2 30382197

[B72] Walter A. Ramos-Murguialday A. Rosenstiel W. Birbaumer N. Bogdan M. Gharabaghi A. (2012). Coupling BCI and cortical stimulation for brain-state-dependent stimulation: Methods for spectral estimation in the presence of stimulation after-effects. Front. Neural Circuits 6, 87. 10.3389/fncir.2012.00087 23162436 PMC3499764

[B74] Wenger N. Moraud E. M. Gandar J. Musienko P. Capogrosso M. Baud L. (2016). Spatiotemporal neuromodulation therapies engaging muscle synergies improve motor control after spinal cord injury. Nat. Med. 22, 138–145. 10.1038/nm.4025 26779815 PMC5061079

[B75] Wolpaw J. R. Birbaumer N. McFarland D. J. Pfurtscheller G. Vaughan T. M. (2002). Brain-computer interfaces for communication and control. Clin. Neurophysiol. 113, 767–791. 10.1016/S1388-2457(02)00057-3 12048038

[B76] Yadav A. P. Li D. Nicolelis M. A. L. (2020). A brain to spine interface for transferring artificial sensory information. Sci. Rep. 10, 900–915. 10.1038/s41598-020-57617-3 31964948 PMC6972753

[B77] Yasui T. Yamaguchi T. Tanabe S. Tatemoto T. Takahashi Y. Kondo K. (2019). Time course of changes in corticospinal excitability induced by motor imagery during action observation combined with peripheral nerve electrical stimulation. Exp. Brain Res. 237, 637–645. 10.1007/s00221-018-5454-5 30536148

[B78] Young D. Willett F. Memberg W. D. Murphy B. Walter B. Sweet J. (2018). Signal processing methods for reducing artifacts in microelectrode brain recordings caused by functional electrical stimulation. J. Neural Eng. 15, 026014. 10.1088/1741-2552/aa9ee8 29199642 PMC5818316

[B79] Zimmermann J. B. Jackson A. (2014). Closed-loop control of spinal cord stimulation to restore hand function after paralysis. Front. Neurosci. 8, 87–88. 10.3389/fnins.2014.00087 24904251 PMC4032985

