# Supplementary material for: Non-invasive brain-spine interface: Continuous control of trans-spinal magnetic stimulation using EEG
Source: Front Bioeng Biotechnol. 2022 Oct 31;10:975037. doi: 10.3389/fbioe.2022.975037 (PMC9659618; doi:10.3389/fbioe.2022.975037)
Supplement: Supplementary file 1 [file DataSheet1.PDF]

Supplementary Material

1 Supplementary Figures

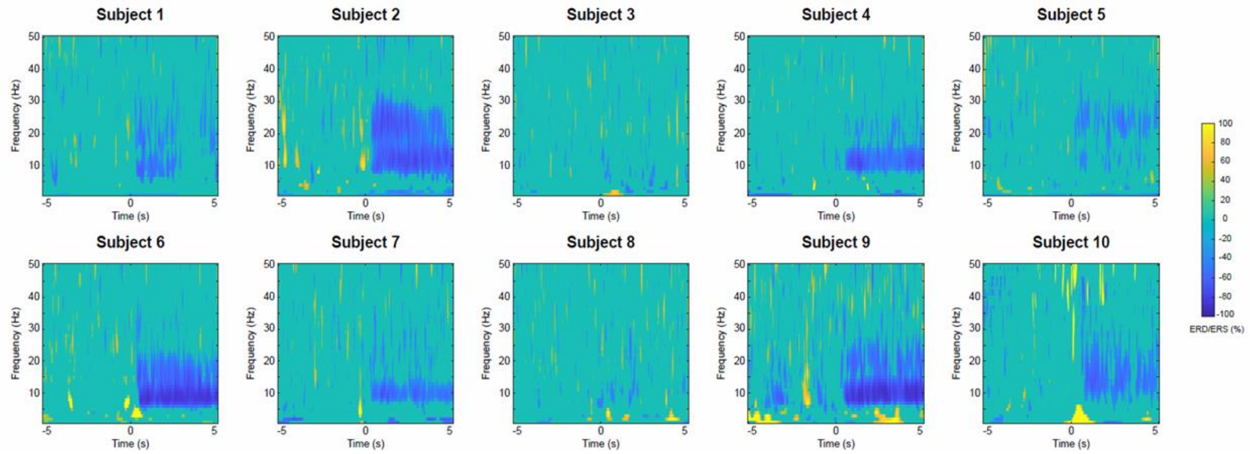

Figure S1. Cortical activation during motor imagery (computed from the screening blocks). Time-frequency maps for each individual, representing the event-related (de)synchronization ERD/ERS (Pfurtscheller and Lopes da Silva, 1999) of the optimized spatial filter (OSF) channel. Time 0 s represents the auditory cue to start the motor imagery.

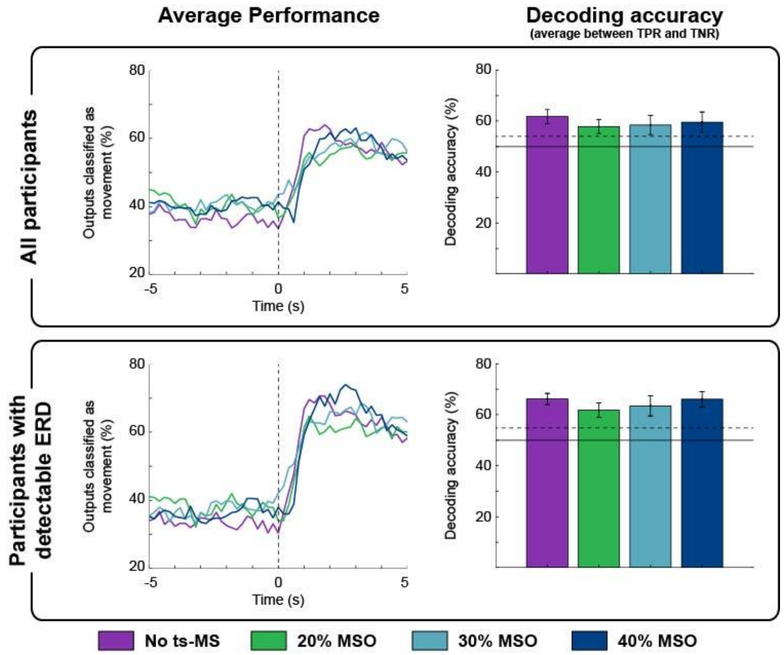

Figure S2. Average response of the classifier for all the stimulation conditions. (Left) Average time-response of the classifier for each stimulation condition. Each line represents the percentage of the outputs classified as motor imagery, averaged over all the participants. Notice that in time 0 s the auditory cue to begin the motor imagery (MI) task is displayed, and outputs prior to t=0 represent false positives, while outputs after t=0 mean true positives. (Right) Decoding accuracy, calculated as the mean between true negative rate (TNR)

in the time interval [-1, -4] s and true positive rate (TPR) in the time interval [1, 4] s. The dashed line shows the confidence interval of the chance level (alpha = 0.05), calculated based on all the test trials, according to (Müller-Putz et al., 2008). Panels show the values averaged for all the participants (top) and for the seven participants with detectable MI-related desynchronization in the alpha frequency band (bottom).

2 Supplementary Movie

Movie S1: Closed-loop guidance of the BSI allowing simultaneous recording of cortical activity and peripheral magnetic stimulation.

3 References

Müller-Putz, G., Scherer, R., Brunner, C., Leeb, R., and Pfurtscheller, G. (2008). Better than random: A closer look on BCI results. *International Journal of Bioelectromagnetism* 10, 52–55.

Pfurtscheller, G., and Lopes da Silva, F. H. (1999). Event-related EEG/MEG synchronization and desynchronization: basic principles. *Clinical Neurophysiology* 110, 1842–1857. doi: 10.1016/S1388-2457(99)00141-8.
